# Hematological and Biochemical Parameters of Subadult Captive Siberian Tigers (*Panthera tigris altaica*)

**DOI:** 10.3390/ani15091299

**Published:** 2025-04-30

**Authors:** Xin Liu, Izhar Hyder Qazi, Haijun Wang, Zhiqiang Han, Xiao Li, Xiuli Zhang, Rui Du, Naiquan Yao, Chao Xu

**Affiliations:** 1 College of Animal Science and Technology, College of Veterinary Medicine, Jilin Agricultural University, Changchun 130118, China; liu6428y@163.com (X.L.); lix9805@163.com (X.L.); durui197101@sina.com (R.D.); 2 Guangdong Provincial Key Lab of Agro-Animal Genomics and Molecular Breeding, College of Animal Science, South China Agricultural University, Guangzhou 510642, China; vetdr_izhar@yahoo.com; 3 Faculty of Biosciences, Shaheed Benazir Bhutto University of Veterinary and Animal Sciences, Sakrand 67210, Pakistan; 4 Jilin Province Northeast Tiger Garden and Jilin Wild Animal Rescue Breeding Center Committee, Changchun 130122, China; wanghaijun117@sina.com; 5 Institute of Zoology, Chinese Academy of Sciences, Beijing 100101, China; hanzhiqiang5113@163.com; 6 College of Veterinary Medicine, Jilin University, Changchun 130062, China; xiuli23@jlu.edu.cn

**Keywords:** conservation, age-specific, endangered species, feline, wildlife

## Abstract

The conservation of Siberian tigers, the apex predators in the Asia–Pacific region, hinges on a thorough understanding of their physiological statuses and adaptive mechanisms. In order to fill a critical gap in our knowledge of this endangered species, we conducted comprehensive analyses of Siberian tiger whole blood and serum using modern hematology and biochemical analyzers. The blood samples were collected from subadult (2–3 years old) Siberian tigers (*n* = 15 for hematological analysis and *n* = 7 for biochemical analysis). In the present study, distinct hematological patterns including an elevated mean platelet volume (MPV) and a higher red cell distribution width coefficient of variation (RDW-CV) were observed, which were possibly triggered by pre-anesthesia resistance movements. Notably, the neutrophil percentage in Siberian tigers was found to be significantly higher than that in domestic cats and Bengal tigers, suggesting species-specific immune adaptations. In addition, serum biochemical analysis provided refined parameter ranges for subadult tigers. Unlike previous studies encompassing a broader age range, our data specifically characterize biochemical profiles in subadult tigers, offering vital baselines for health monitoring and conservation strategies. These results not only enhance our understanding of the biology of Siberian tigers but also provide scientific support for targeted conservation efforts, fostering population recovery and ecological balance in their natural habitats.

## Simple Summary

The Siberian tigers are the apex predators in the Asia–Pacific region. This species has been listed as “Endangered” on the IUCN Red List since 1986. Establishing standardized hematological and biochemical parameters is critical for monitoring the health of wild populations, particularly in the conservation of endangered species. In order to understand these tigers’ physiological statuses and adaptive mechanisms, in this report, we provide comprehensive blood and biochemical profiles of subadult Siberian tigers. The findings of the present report provide a reasonable foundation for health monitoring, targeted conservation efforts, and the population recovery of Siberian tigers in their natural habitats.

## 1. Introduction

The Siberian tiger, serving as a pivotal flagship species in wildlife conservation, has been categorized as “Endangered” on the IUCN Red List since 1986 [1,2]. Unfortunately, its vulnerable status still persists despite being the object a considerable amount of global attention and benefiting from collaborative conservation efforts that have facilitated a population resurgence, increasing from roughly 30–40 individuals in the 1940s to an estimated 523–540 in 2015 [3]. The trajectory of Siberian tiger population growth is impeded by a multifaceted array of challenges, including habitat degradation and fragmentation, diminished genetic diversity, and a declining prey base [4,5]. Moreover, in recent years, infectious diseases such as canine distemper virus and parasitic (e.g., roundworm) infestations have emerged as significant threats to the health and resilience of these populations [6,7]. If we are to safeguard this majestic species, a profound understanding of their basic physiological and health statuses is crucial for devising effective conservation and management strategies [8]. Hematology, as a cardinal tool, plays a paramount role in evaluating the health metrics of these wilds animals and exploring the physiological statuses of their organs’ functions [9,10,11]. Comprehensive analysis of blood constituents including cellular components, plasma, and biochemical constituents provides invaluable insights useful for disease diagnosis, nutritional assessments, and reproductive status determination, including pregnancy detection [12,13]. To assess the overall health of these animals, meticulous examination of their hematological and biochemical parameters is important. Hematology, in particular, sheds light on an organism’s capacity for oxygen transportation, immune competence, and homeostatic maintenance, with a particular focus on the composition and proportion of immune cells, which are instrumental in defense mechanisms and maintaining an overall physiological equilibrium [14]. Biochemical analysis offers insights into the functional integrity of various organs and aids in identifying any metabolic imbalance or nutritional deficiencies that may compromise the well-being of tigers [15]. The application of whole-blood analysis to discern deviations from established normal values in specific hematological or biochemical parameters is an indispensable practice [16]. However, prior to detecting such anomalies in these animals, it is important to establish species-specific (considering age range and sex) and baseline parameters. These benchmarks serve as critical reference points for exploring the origins of deviations and informing potential intervention strategies [17]. Through the establishment and careful monitoring of these parameters, conservationists and researchers can more effectively work towards tracking and improving the health status of Siberian tigers. These efforts can help sustain Siberian tiger populations in the wild. In this study, we evaluated the hematological and biochemical parameters of subadult Siberian tigers, laying a foundation for the conservation of this endangered species and the health monitoring of wild populations.

## 2. Material and Methods

### 2.1. Animal Source and Management

All samples were sourced from a captive population of Northeast tigers at a zoo (Northeast Tiger Garden) in Jilin Province, China. The experimental protocol was approved by the Animal Welfare and Ethics Committee of Jilin Province Northeast Tiger Garden (DF23JF231), ensuring compliance with ethical standards for animal research. In this study, hematological and biochemical parameters were measured in a total of 15 tigers, with seven males and eight females. All tigers were between two and three years of age. The husbandry and management of the tigers were carried out according to the Technical Code of Feeding and Management for Wild Animals—Siberian tiger (LY/T 2199-2013). In brief, the diet of the captive tigers primarily consisted of beef, mutton, and chicken, supplemented with milk, eggs, cod liver oil, vitamins, trace elements, and minerals. All feed components were stored separately prior to feeding to minimize the risk of cross-contamination. Additionally, to simulate the fasting periods the animals might experience in the wild, the tigers were subject to one day of complete fasting and one day of partial fasting each week. Prior to any procedures, the tigers were subjected to routine clinical physical examinations to ensure they were healthy. Only those exhibiting no clinical signs of illness were selected for further testing. Additionally, to standardize the physiological states of the tigers at the time of sampling, all tigers were fasted for 24 h and deprived of water for 12 h before being restrained. Anesthesia was induced using compound ketamine injection (2 mL containing 0.3 g of ketamine hydrochloride, 0.3 g of dimethy thiazide hydrochloride, and 1 mg of phenethylpiperate hydrochloride; Jiangsu Zhongmu Beikang Pharmaceutical Co., Ltd., Taizhou, China), administered at a dose of 0.02 mL per kilogram of body weight.

### 2.2. Sample Collection

With the tigers under general anesthesia, we collected blood samples from the saphenous vein of each tiger. The blood was aseptically collected into BD Vacutainer^®^ K_2_EDTA Blood Collection Tubes (Becton Dickinson S.p.A., Milano, Italy) and BD Vacutainer^®^ serum Blood Collection Tubes (Becton Dickinson S.p.A., Milano, Italy). After collection, the K_2_EDTA tubes were thoroughly mixed by inverting them, whereas the serum tubes were left at room temperature for 30 min and then centrifuged at 3000 rpm for 10 min to separate the serum, which was subsequently stored at 4 °C. All samples were processed and analyzed in a single batch after the completion of the collection and separation steps to minimize potential variations in the results. This standardized protocol was adopted to ensure the integrity and reproducibility of the blood samples were sufficient for subsequent biochemical and physiological assays. Note: Due to administrative limitations and in order to adhere to the 3Rs principles of animal welfare and minimize handling stress, we decided to collect duplicate samples (for serum biochemistry analysis) from only seven tigers.

### 2.3. Parameter Measurement

Hematological parameters were assessed using a ProCyte Dx Hematology Analyzer (IDEXX Laboratories, Westbrook, ME, USA) in accordance with the manufacturer’s protocol [18]. Prior to its use, the instrument underwent routine maintenance, calibration, and quality control to ensure its optimal performance. Blood samples were thoroughly mixed via gentle inversion before being analyzed. The following hematological parameters were measured: red blood cell concentration (RBC), hemoglobin (HGB), hematocrit (HCT), mean corpuscular volume (MCV), mean corpuscular hemoglobin (MCH), mean corpuscular hemoglobin concentration (MCHC), red cell distribution width standard deviation (RDW-SD), red cell distribution width coefficient of variation (RDW-CV), platelet (PLT), platelet distribution width (PDW), mean platelet volume (MPV), platelet concentration (PCT), white blood cell concentration (WBC), neutrophil concentration (NEUT), lymphocyte concentration (LYM), monocyte concentration (MONO), eosinophil concentration (EO), and basophil concentration (BASO). Serum samples were separated and stored at 4 °C on the same day of collection and analyzed within six hours. Serum biochemical profiles were evaluated using the VETSCAN VS2 Chemistry Analyzer (Zoetis, Parsippany, NJ, USA), which quantified the following analytes: albumin (ALB), alkaline phosphatase (ALP), alanine aminotransferase (ALT), amylase (AMY), total bilirubin (TBIL), blood urea nitrogen (BUN), calcium (CA), phosphorus (PHOS), creatinine (CRE), glucose (GLU), sodium (Na^+^), potassium (K^+^), total protein (TP), and globulin (GLOB).

### 2.4. Statistical Analysis

Shapiro–Wilk normality tests were employed to assess the normality of sex variables. All data are expressed as minimums, maximums, means, and standard deviations. Statistical analysis was conducted using *t*-tests, with *p* < 0.05 considered statistically significant.

## 3. Results

**Figure 1:**
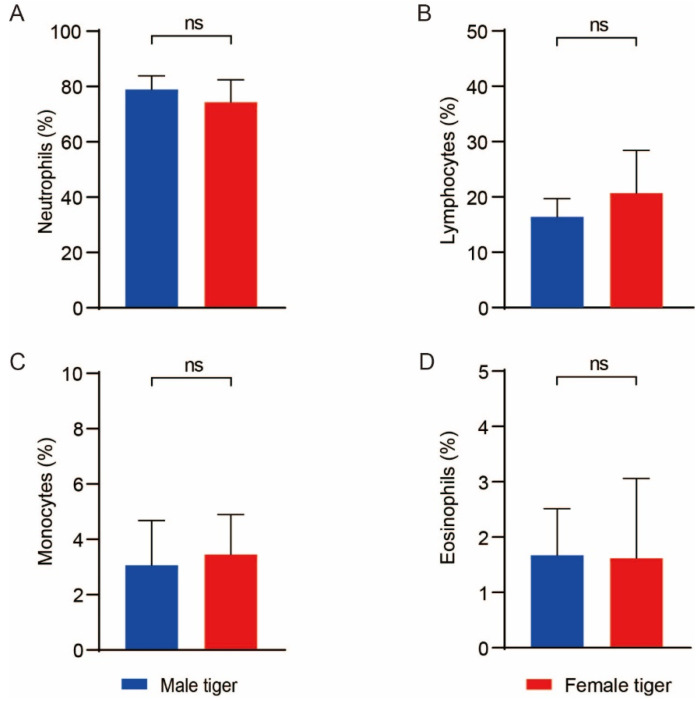
Comparison of white blood cell percentages in the peripheral blood of male and female subadult Siberian tigers: neutrophils (**A**), lymphocytes (**B**), monocytes (**C**), and eosinophils (**D**). ns: *p* > 0.05.

Following the clinical examination, 15 out of 17 tigers were deemed clinically healthy. Hematological and serum biochemical parameters were measured using EDTA samples from 15 tigers and serum samples from 7 tigers. The results are presented as the ranges of different physiological parameters and means ± standard deviations for captive subadult Siberian tigers (Table 1). No significant differences in hematological parameters were observed between sexes (*p* > 0.05). In addition, compared to the previous International Species Information System (ISIS) data (covering the period from 8 days to 3 years), no significant differences were observed with respect to the hematological and biochemical parameters in the population of Siberian tigers studied in the present study [19]. The cellular composition of the blood of the subadult Siberian tigers was found to be similar to that of other animals, primarily consisting of red blood cells (RBCs) and white blood cells (WBCs). The distribution of WBCs was as follows (see Figure 1): a neutrophil concentration from 64.3 to 88.2% (mean ± SD: 76.83 ± 6.73%), a lymphocyte concentration from 10.33 to 31.9% (18.40 ± 5.98%), a monocyte concentration from 1.2 to 5.7% (3.25 ± 1.49%), and an eosinophil concentration from 0 to 4.4% (1.43 ± 1.48%). Due to the limited number of serum biochemical samples collected from subadult Siberian tigers in the present study, only the range of biochemical parameters was statistically analyzed (Table 2).

## 4. Discussion

The conservation of the Siberian tiger, the largest feline species in the Asia–Pacific region, is hindered by significant challenges [21,22]. Assessing the hematological and biochemical parameters of wild animals like tigers is of primary importance in order to understand their fundamental biological characteristics, strengthen disease diagnosis and prevention measures, optimize reproductive management practices, advance environmental adaptability studies, drive genetic diversity analyses, and inform the development of ecological conservation strategies [21,23,24,25,26]. This basic research could provide a valuable scientific evidence base for efforts aimed at protecting the Siberian tiger, promoting their population recovery and maintaining ecological balance. Due to the unique behavioral and ecological adaptations of wild animals, capturing them in their natural habitats to obtain blood, urine, or other biological samples is extremely difficult [27,28]. Research on the hematological and biochemical parameters of tiger populations is limited, especially for different age groups. In this study, the hematological parameters of 15 subadult Siberian tigers and the biochemical parameters of 7 subadult Siberian tigers were analyzed, with the aim of contributing to the development of a comprehensive physiological parameter database for tigers.

The results of this study are consistent with the ISIS physiological reference intervals for captive wildlife, specifically for WBC, LYM, MON, NEU, EOS, RBC, HCT, HGB, MCH, and PLT. There were no significant differences in blood physiological parameters between male and female tigers (*p* > 0.05). The MPV and RDW-CV values in our study are higher than those reported by Liu et al. [20]. A high MPV often indicates increased production of new platelets, which suggests enhanced or recovering hematopoietic function. RDW-CV is a parameter used in routine blood tests that reflects the variability in the volume of red blood cells in peripheral blood. An elevated RDW-CV typically indicates that the RBCs examined are of varying sizes; i.e., there is increased heterogeneity in red blood cell volume. We believe that the increase in these parameters may be related to the resistance movements of animals before anesthesia. 

Hilberg et al. [29] reported that moderate exercise can increase platelet reactivity and the formation of platelet–leukocyte complexes, which may contribute to higher MPV measurements. The elevated levels of MPV and RDW-CV in this study may be related to handling stress and the effects of anesthesia, which are common factors in wild animal research, particularly for these large and aggressive carnivores. However, there are currently no plausible explanations supporting this notion. Studies on humans have shown that an elevated RDW may be related to aging, race, and physical activity [30]. We know that the immobilization of wild animals is difficult to achieve with physical restraint tools and taming alone, especially for these aggressive large felid species. The immobilization of these animals for invasive procedures or sample collection must be achieved with the help of chemical means. Therefore, the physiological responses of wild animals to anesthesia are unavoidable, and as a norm, these responses can only be appropriately managed by monitoring changes in body temperature, respiratory and heart rates, gas exchange, and acid–base balance.

Unlike previous studies, we focused on the proportions of five types of WBCs: neutrophils, eosinophils, basophils, lymphocytes, and monocytes. For comparison, in Bengal tigers, the proportions of neutrophils, lymphocytes, monocytes, and eosinophils are 57–75% (60 ± 5.08%), 18–35% (30 ± 4.56%), 2–6% (5 ± 1.21%), and 2–6% (4 ± 1.30%), respectively [31]. In cats, the proportions of neutrophils, lymphocytes, monocytes, and eosinophils are 45–64%, 27–36%, 0–5%, and 0–4%, respectively [32]. We found that the neutrophil percentage in Siberian tigers was higher than that in domestic cats and Bengal tigers [31], while the other indicators were within similar ranges. These differences may be attributable to variations in sample size, age, or inter-breed differences. Another possible explanation is the presence of a common adaptive mechanism in the mammalian immune system. Although it remains unclear how different pools of neutrophils are initially established and how they attain a dynamic equilibrium, it is clear that under certain stress conditions, the demarginated pool may contribute significantly to the circulating pool of neutrophils [33]. During anesthesia, excitement and movement can trigger the release of adrenaline and noradrenaline [34,35]. These hormones interact with corresponding receptors on immune cells, affecting their development, trafficking, and function, ultimately resulting in a shift of neutrophils from the marginating to the circulating pool [36]. Overall, due to the low sensitivity of hematological tests, some indicators may be less accurate, such as eosinophils and basophils. However, the relative proportions of the various WBCs are consistent: neutrophils are the most abundant, followed by lymphocytes, monocytes, eosinophils, and basophils. In addition to hematological analyses, we also measured the serum biochemical parameters in seven Siberian tigers. The results were consistent with the tiger data for 8-day to 3-year-old tigers from the previous ISIS records [19]. Notably, our parameter ranges are narrower compared to those reported by others [20,23], including with respect to ALB, ALP, ALT, AMY, TBIL, BUN, CA, PHOS, CRE, GLU, Na^+^, K^+^, and TPGLOB. This may be attributed to the fact that all the tigers in our study were subadults (2–3 years old), whereas other studies included animals aged 2–9 years. Similar trends in terms of glucose, GLU, BUN, CRE, TP, ALT, and ALP were observed in a study of 12 tigers aged 1–9 years [15]. Our data provide a more specific reflection of the biochemical parameter ranges for subadult tigers (2–3 years old), laying a foundation for future health studies on this age group.

Overall, this study provides comprehensive data on the hematological and biochemical parameters of a small population of subadult Siberian tigers. Our study was hindered by its limited sample size and datasets, which are common and acknowledged constraints in ecological and wildlife research [37]. Additionally, the lack of a blood smear evaluation limited the data collected in the present analysis, so we are unable to comment on the presence of neutrophil bands or the morphology of RBCs and WBCs. Therefore, we propose conducting more adequately powered studies to enhance the utility of the data presented in the present study.

## 5. Conclusions

In this report, we provide a detailed description of the hematological and biochemical parameters of subadult Siberian tigers. Despite inherent variations due to biological, methodological, and analytical factors, the parameters obtained in this research serve as valuable references for physiological studies on Siberian tigers. This work lays the foundation for developing a comprehensive hematological database for Siberian tigers and determining their physiological blood indicators while also guiding future immunological research on this species. Collectively, these findings provide valuable data support for advancing both scientific research and conservation efforts aimed at protecting Siberian tigers.

## Figures and Tables

**Table 1:** Comparison of hematological parameters between subadult Siberian tigers and the ISIS reference intervals.

Hematology	Male (*n* = 7)		Female (*n* = 8)		Total (*n* = 15)		Reference Intervals
	Range	(Mean ± SD)	Range	(Mean ± SD)	Range	(Mean ± SD)	
RBC (M/μL)	7.06–8.01	7.49 ± 0.34	7.09–8.79	7.77 ± 0.60	7.06–8.79	7.63 ± 0.50	3.64–10.10
HGB (g/dL)	13.1–16.0	14.24 ± 1.00	13.4–16.5	14.54 ± 1.23	13.1–16.5	14.4 ± 1.10	6.5–18.7
HCT (%)	39.10–45.35	42.49 ± 2.78	38.70–48.39	43.07 ± 3.18	38.70–48.39	42.80 ± 2.91	21.0–54.7
MCV (fL)	48.8–59.3	56.69 ± 3.70	49.2–58.0	55.51 ± 2.77	48.8–59.3	56.06 ± 3.17	33.6–92.3
MCH (pg)	17.4–20.9	19.04 ± 1.07	17.3–19.7	18.74 ± 0.75	17.3–20.9	18.88 ± 0.90	7.8–30.2
MCHC (g/dL)	31.5–35.9	33.56 ± 1.61	31.8–35.1	33.76 ± 1.17	31.5–35.9	33.67 ± 1.34	24.1–50.0
RDW-SD (fL)	38.4–46.1	41.83 ± 3.57	39.1–44.5	42.80 ± 1.98	38.4–46.1	42.35 ± 2.77	
RDW-CV (%)	19.8–23.9 *	21.91 ± 1.88 *	19.6–25.7 *	21.71 ± 2.40 *	19.6–25.7 *	21.81 ±2.10 *	12–17.5
PLT (K/μL)	118–302	245.00 ± 60.93	74–385	203.50 ± 94.52	74–385	222.87 ± 80.73	101–987
PDW (fL)	22.6–25.2	23.77 ± 1.32	7.6–23.0	19.30 ± 6.59	7.6–25.2	20.98 ± 5.54	
MPV (fL)	13.7–17.6 *	15.34 ± 1.31 *	14.0–16.0 *	14.95 ± 0.83 *	13.7–17.6 *	15.13 ± 1.06 *	5.5–10.5
PCT (%)	0.18–0.5	0.38 ± 0.10	0.12–0.55	0.30 ± 0.13	0.12–0.55	0.34 ± 0.12	
WBC (K/μL)	8.88–15.46	12.13 ± 2.56	8.56–14.63	10.71 ± 1.92	8.56–15.46	11.37 ± 2.28	4.5–26.4
NEUT# (K/μL)	6.04–12.56	9.13 ± 2.55	6.85–11.86	8.46 ± 1.69	6.04–12.56	8.77 ± 2.08	0–7.760
LYMPH# (K/μL)	1.24–3.16	2.41 ± 0.77	0.88–2.40	1.76 ± 0.44	0.88–3.16	2.06 ± 0.68	0.008–9.450
MONO# (K/μL)	0.2–0.88	0.43 ± 0.23	0.13–0.52	0.32 ± 0.16	0.13–0.88	0.37 ± 0.20	0.000–2.431
EO# (K/μL)	0–0.39	0.15 ± 0.14	0.08–0.29	0.18 ± 0.07	0–0.39	0.16 ± 0.11	0.000–1.215
BASO# (K/μL)	-	-	-	-	-	-	3.64–10.10

Note: The reference intervals from ISIS include data from tigers aged 8 days to 3 years and cover the following parameters: RBC, HGB, HCT, MCV, MCH, MCHC, PLT, WBC, NEUT, LYMPH, MONO, and EO. The reference intervals for RDW-CV and MPV are from Liu et al. [20]. * indicates the hematological parameters of subadult Siberian tigers in this study that differ from the reference intervals from the study by Liu et al. [20].

**Table 2:** Comparison of biochemical parameters between subadult Siberian tigers and the ISIS reference intervals.

	Range	Mean ± SD	Reference Intervals
ALB (g/dL)	4–4.3	4.14 ± 0.13	2.5–5.1
ALP (U/L)	42–97	68.13 ± 19.27	8–483
ALT (U/L)	56–83	64.50 ± 8.43	13–118
AMY (U/L)	478–595	506.50 ± 37.05	160–2133
TBIL (mg/dL)	0.3–0.3	0.30 ± 0.00	0–1
BUN (mg/dL)	20–25	22.75 ± 1.91	11–50
CA (mg/dL)	10.7–12	11.45 ± 0.44	8.4–13.2
PHOS (mg/dL)	4.5–7.7	6.49 ± 1.21	3.5–11.3
CRE (mg/dL)	1.4–3	2.05 ± 0.62	0.3–4.3
GLU (mg/dL)	151–237	191.50 ± 32.38	0–355
Na+ (mmo1/L)	150–157	154.63 ± 2.33	134–165
K+ (mmo1/L)	4–4.5	4.29 ± 0.16	3.5–7.6
TP (g/dL)	6.8–7.6	7.21 ± 0.27	4.5–8.7
GLOB (g/dL)	2.6–3.6	3.06 ± 0.31	1.8–5.2

Note: The reference intervals from ISIS, including tiger age datasets from 8 days to 3 years of age, were used for comparison.

## Data Availability

The raw data supporting the conclusions of this article will be made available by the authors upon request.
